# Cochlear activity in silent cue-target intervals shows a theta-rhythmic pattern and is correlated to attentional alpha and theta modulations

**DOI:** 10.1186/s12915-021-00992-8

**Published:** 2021-03-16

**Authors:** Moritz Herbert Albrecht Köhler, Gianpaolo Demarchi, Nathan Weisz

**Affiliations:** 1 Centre for Cognitive Neuroscience, University of Salzburg, Hellbrunner Straße 34, 5020 Salzburg, Austria; 2 Department of Psychology, University of Salzburg, Hellbrunner Straße 34, 5020 Salzburg, Austria

**Keywords:** Cochlea, Auditory periphery, Efferent, MEG, Otoacoustic, Alpha, Theta, Attention, Rhythmicity, Intermodal

## Abstract

**Background:**

A long-standing debate concerns where in the processing hierarchy of the central nervous system (CNS) selective attention takes effect. In the auditory system, cochlear processes can be influenced via direct and mediated (by the inferior colliculus) projections from the auditory cortex to the superior olivary complex (SOC). Studies illustrating attentional modulations of cochlear responses have so far been limited to sound-evoked responses. The aim of the present study is to investigate intermodal (audiovisual) selective attention in humans simultaneously at the cortical and cochlear level during a stimulus-free cue-target interval.

**Results:**

We found that cochlear activity in the silent cue-target intervals was modulated by a theta-rhythmic pattern (~ 6 Hz). While this pattern was present independently of attentional focus, cochlear theta activity was clearly enhanced when attending to the upcoming auditory input. On a cortical level, classical posterior alpha and beta power enhancements were found during auditory selective attention. Interestingly, participants with a stronger release of inhibition in auditory brain regions show a stronger attentional modulation of cochlear theta activity.

**Conclusions:**

These results hint at a putative theta-rhythmic sampling of auditory input at the cochlear level. Furthermore, our results point to an interindividual variable engagement of efferent pathways in an attentional context that are linked to processes within and beyond processes in auditory cortical regions.

**Supplementary Information:**

The online version contains supplementary material available at 10.1186/s12915-021-00992-8.

## Background

Cognitive processing of sensory stimuli is capacity limited. Hence, attentional processes are required to prioritize cognitive resources on task- or context-relevant stimuli. On a neural level, responses to attended stimuli are enhanced, while responses to unattended and distracting stimuli are diminished [1, 2]. These effects have been mainly established on a cortical level [3, 4]; however, it is less clear to what extent selective attention modulates subcortical activity [5]. For the auditory system, this dispute extends down to the level of the cochlea [6–8].

Indeed, cochlear processes can be modulated via direct and mediated (by the inferior colliculus) projections from the auditory cortex to the superior olivary complex (SOC). The SOC finally innervates the outer hair cells (OHC) that are essential for cochlear amplification and fine tuning of the basilar membrane [9]. The architecture of the efferent auditory system would—in principle—enable the auditory cortex to modulate cochlear processes [10].

An increasing number of studies support this notion by measuring otoacoustic emissions (OAE; [11–13]) or cochlear microphonics [14]. However, the described effects are restricted to sound-evoked responses, are small, and sometimes contradictory [15, 16]. Furthermore, the attention research on cortical and cochlear processes has been conducted largely independently (see [13, 17, 18] for exceptions). In summary, it remains unclear whether and how attention modulates cochlear processes during silent periods and how these peripheral processes are linked to cortical processes.

We applied an established intermodal (audiovisual) selective attention task and simultaneously measured activity from different levels of the auditory system, to advance our knowledge in this area. To stay as close as possible to previous magnetoencephalography and electroencephalography (M/EEG) works in this domain [17, 18], we decided to record sounds within the ear canal during silent cue-target intervals. This “ongoing otoacoustic activity” (OOA) allows for an unbiased measurement of cochlear modulations by cortical attention processes, since undesired sound-evoked cochlear changes are circumvented [19].

Given that attentional modulations of cortical oscillations are mostly found at low frequencies (< 30 Hz), we decided to use a similar analysis approach for the OOA-signal as Dragicevic et al. [20], an approach that allows us to investigate oscillatory cochlear activity at the same frequencies as cortical activity occurs. Further, genuine periodic components (peaks) of the OOA-signal were computed for the OOA [21]. Replicating an established finding from several previous studies [13, 22, 23], we show strong attentional modulation of visual cortical alpha activity. More importantly, we illustrate a rhythmic modulation of cochlear activity in the theta frequency range. While this theta activity was generally present independently of attentional focus, it was strongly amplified when attending to the auditory modality. Interestingly, this attentional amplification of cochlear activity is inversely correlated with attentional alpha and theta effects at the cortical level across participants.

## Results

### Behavioral results

Performance in terms of accuracy was similar for both conditions and in general very high, underlining the compliance of the participants during the experiment. The average accuracy was *M* = 93.19% (*SD* = 7.46%) for the auditory task and *M* = 92.89% (*SD* = 7.65%) for the visual task. The accuracies of the two conditions did not differ significantly (*t*_(26)_ = 0.378, *p* = 0.709). The generally high levels of accuracy suggest a ceiling effect for this behavioral index. This can be explained by the fact that this study did not utilize threshold-level stimuli. Analysis of reaction times revealed significantly longer reaction times for the auditory (*M* = 552.5 ms, *SD* = 181.5 ms) compared to the visual task (*M* = 493.3 ms, *SD* = 177.9 ms; *t*_(26)_ = 3.7302, *p* = 0.0009).

### OOA at theta rhythm is modulated by intermodal attention

Typical oscillatory activity of the brain is pronounced in a frequency band of 1–80 Hz, whereas otoacoustic activity is found at much higher frequencies (500–4000 Hz). As the aim of this experiment is to study the effects of cortical top-down modulations on OOA, we applied the Hilbert transform to extract the amplitude modulation for frequencies typical of ongoing cortical oscillations. To avoid a stronger influence of the lower sound frequencies and to create a representation of the cochlea’s frequency response, the otoacoustic signal was bandpass filtered between 1000 and 2000 Hz in 10 Hz steps with a window size of ± 30 Hz. The power spectral densities (PSD) of the 201 bandpass windows were then concatenated to create a representation of the amplitude modulation between 1000 and 2000 Hz of the cochlea’s frequency response.

**Fig. 1:**
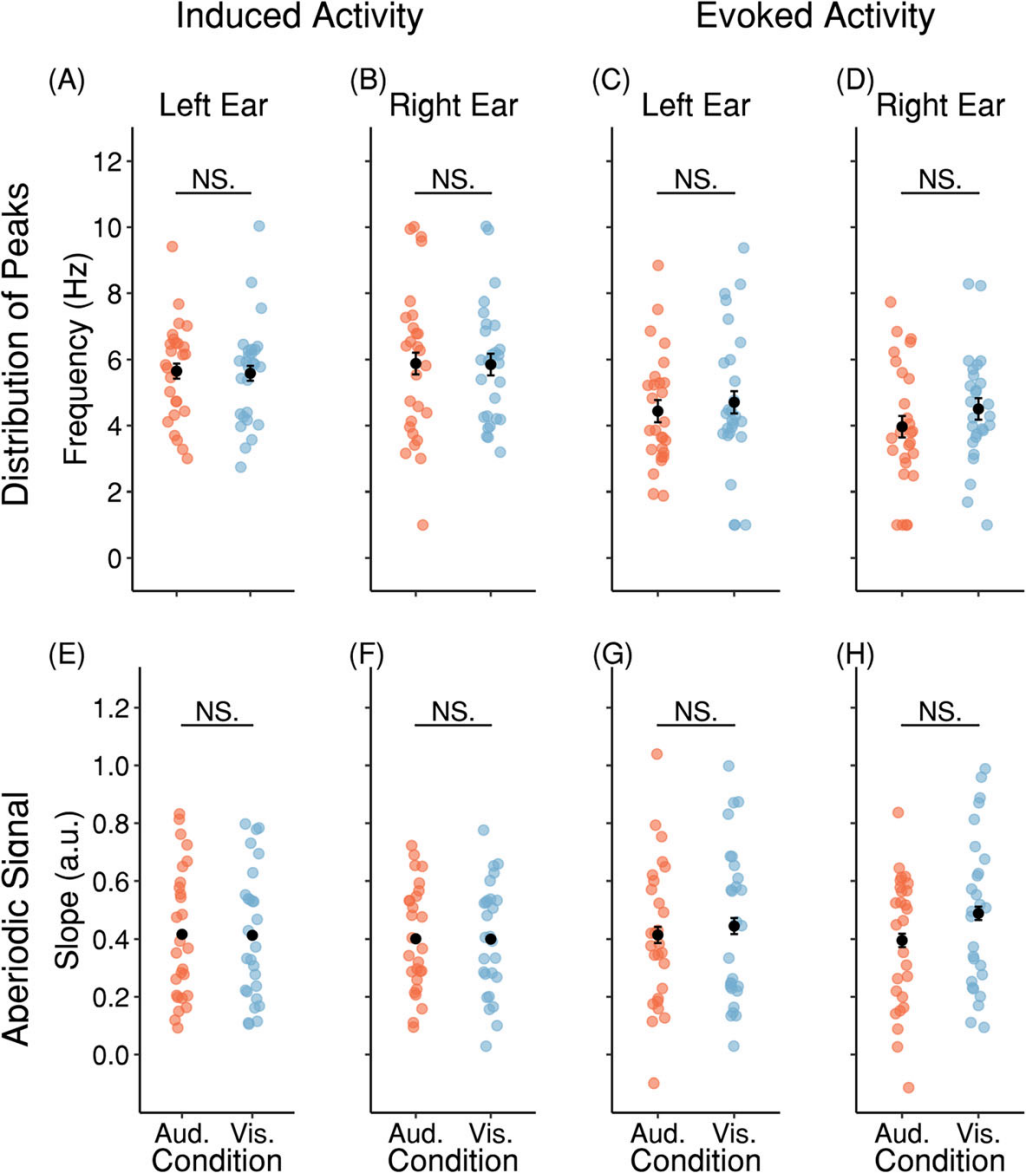
Peak analysis of OOA by FOOOF shows theta rhythmicity of cochlear activity. **a**–**d** Distribution of peaks for induced and evoked activity in the left and right ear for each subject and condition. **e**–**h** Slopes of the aperiodic signal for induced and evoked activity in the left and right ear for each subject and condition. The black dots and error bars represent the mean and SEM (corrected for within-subject designs; see [24])

In a first step, we parameterized induced oscillatory modulations of OOA during the silent cue-target interval. We used the FOOOF-toolbox to differentiate between genuine oscillatory contributions from aperiodic 1/f changes. In all subjects, a peak could be found at low (< 11 Hz) frequencies with a clustering around ~ 5–6 Hz. However, it has to be noted that for a number of subjects more than one peak was identified below 11 Hz. For the Attend Auditory condition, the average peak frequency was at 5.65 Hz (*SD* = 1.48) for the left and 5.88 Hz (*SD* = 2.33) for the right ear. For the Attend Visual condition, the average peak frequency was at 5.58 Hz (*SD* = 1.57) for the left and at 5.85 Hz (*SD* = 1.83) for the right ear. Which modality was attended to had no statistically significant impact on the peak frequencies in both ears (left: *t*_(26)_= 0.2068, *p* = 0.9462; right: *t*_(26)_ = 0.0681, *p* = 0.9462; FDR-corrected (false detection rate)). For the Attend Auditory condition, the average slope was at 0.416 (*SD* = 0.229) for the left and 0.401 (*SD* = 0.184) for the right ear. For the Attend Visual condition, the average slope was at 0.413 (SD = 0.226) for the left and 0.400 (SD = 0.192) for the right ear. We found no statistically significant effect of modality for slopes in both ears (left: *t*_(26)_ = 0.9462, *p* = 0.6503; right: *t*_(26)_ = 0.1107, *p* = 0.9462; FDR-corrected). Figure 1a, b show subjects’ individual peak frequencies and Fig. 1e, f the slope for aperiodic components (“1/f noise”). In order to test if the identified peaks are significant components of the respective PSD, we evaluated for every PSD if the power at the peak frequency is a significant outlier of the distribution of the power at frequencies that were not identified as peaks. For this purpose, we calculated Dixon’s *Q* tests for every PSD except 1 that did not fulfill the requirements of Dixon’s *Q* test. In 104 of the 107 tested PSDs, the power at the peak frequency was a significant outlier. An exact binomial test indicated that the proportion of found significant outliers of 0.97 was higher than the expected 0.50, which would be expected if the power at the peak frequencies were outliers by chance (*p* < 0.0001, two-sided). Moreover, we performed Kolmogorov-Smirnov tests to test for uniformity on the peak frequencies for every ear and condition. The percentage of peak frequencies for the left ear and Attend Auditory condition (*D*_(26)_ = 9.2347, *p* < 0.0001) and the percentage of peak frequencies for the left ear and Attend Visual (*D*_(26)_ = 9.2486, *p* < 0.0001) were both significantly different from uniformity, indicating that the peak frequencies were not uniformly distributed in both conditions. The same holds true for the right ear (Attend Auditory: *D*_(26)_ = 9.4619, *p* < 0.0001; Attend Visual: *D*_(26)_ = 9.3502, *p* < 0.0001). While this analysis overall points to a theta-rhythmic modulation of cochlear activity in a silent cue-target interval, the range (1–10.03 Hz) of these peaks suggests a rather high interindividual variability.

Subsequently, we were interested if the ~ 6 Hz component was phase aligned given that the target was temporally predictable. We calculated evoked power in the same way as described above and then used the FOOOF-toolbox to extract periodic components. For the Attend Auditory condition, the average peak frequency was at 4.44 Hz (*SD* = 1.70) for the left and 3.97 Hz (*SD* = 1.90) for the right ear. For the Attend Visual condition, the average peak frequency was at 4.71 Hz (SD = 2.17) for the left and 4.50 Hz (SD = 1.66) for the right ear. Which modality was attended to had no statistically significant impact on the peak frequencies in both ears (left: *t*_(26)_ = − 0.5628, *p* = 0.9462; right: *t*_(26)_ = − 1.1651, *p* = 0.9462; FDR-corrected). For the Attend Auditory condition, the average slope was at 0.414 (*SD* = 0.249) for the left and 0.395 (*SD* = 0.230) for the right ear. For the Attend Visual condition, the average slope was at 0.445 (SD = 0.271) for the left and 0.489 (SD = 0.265) for the right ear. We found no statistically significant effect of modality for slopes in both ears (left: *t*_(26)_ = − 0.7814, *p* = 0.9462; right: *t*_(26)_ = − 2.855, *p* = 0.0664; FDR-corrected). Figure 1c, d show subjects’ individual peak frequencies and Fig. 1g, h the slope for aperiodic components (“1/f noise”). Tests for uniformity analogue to the ones used for the induced signal uncovered that the percentages of peak frequencies for both ears and attention conditions were significantly different from uniformity (left ear, Attend Auditory: *D*_(26)_ = 8.2005, *p* < 0.0001; left ear, Attend Visual: *D*_(26)_ = 9.1565, *p* < 0.0001; right ear, Attend Auditory: *D*_(26)_ = 7.0971, *p* < 0.0001; right ear, Attend Visual: *D*_(26)_ = 7.8705, *p* < 0.0001). Moreover, as for induced power we performed Dixon’s *Q* tests for every PSD except for 22 that did not fulfill the requirements of Dixon’s *Q* test. In 86 of the 86 tested PSDs, the power at the peak frequency was a significant outlier. An exact binomial test indicated that the proportion of found significant outliers of 1.00 was higher than the expected 0.50, which would be expected if the power at the peak frequencies were outliers by chance (*p* < 0.0001, two-sided). Thus, we assume that the identified peaks are significant components of their respective PSDs. Finally, we were interested if the phase of the evoked oscillation is different between modalities and ears. With this in mind, we calculated FDR-corrected circular common median tests. The results showed no significant difference for both ear and modality (ear: *P*_(26)_ = 0.3068, *p* = 0.5860; modality: *P*_(26)_ = 0.2967, *p* = 0.5860; FDR-corrected). The results suggest that the evoked ~ 4 Hz component is not modulated by attentional focus and the same for both ears. Moreover, we tested if the induced and evoked components were different in frequency. A two-sided *t* test revealed that the frequency of the induced ~ 6 Hz component was significantly higher than that of the evoked ~ 4 Hz component (*t*_(26)_ = 4.4373, *p* = 0.0001). The result suggests that the induced ~ 6 Hz component is different from the evoked one. Thus, we assume that the ~ 6 Hz component is not consistent in phase for the cue-target interval.

**Fig. 2:**
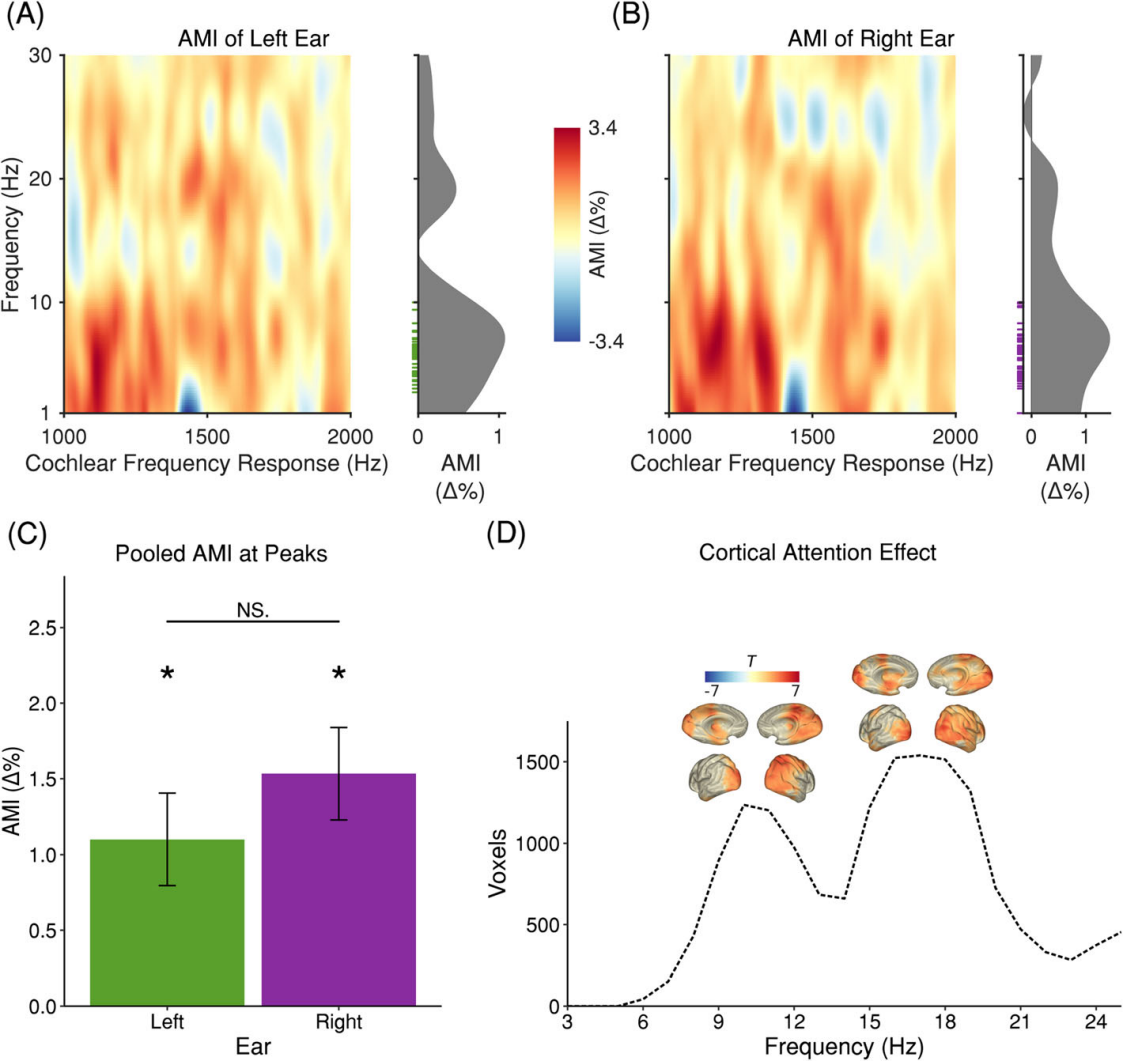
Power analysis of OOA shows enhanced low-frequency power for auditory attention. Power analysis of cortical activity reveals enhanced alpha and beta power for auditory attention. **a**, **b** AMI of the cochlear frequency response for the left and right ear. The *x*-axis represents otoacoustic activity at sound frequencies from 1000 to 2000 Hz. The *y*-axis represents the frequency range of the FFT. On the right of each subplot, the OOA-AMI averaged over sound frequencies from 1000 to 2000 Hz is shown. The green and violet ticks illustrate the distribution of subjects’ peak frequencies from Figs. 1a, b. **c** OOA-AMI averaged over sound frequencies from 1000 to 2000 Hz and the range of subjects’ peak frequencies (3–10 Hz for the left and 1–10 Hz for right ear). The OOA-AMI is significantly higher for the Attend Auditory condition in the left (*t*_(26)_ = 2.4701, *p* = 0.0204) and the right (*t*_(26)_ = 2.3881, *p* = 0.0245) ear. There was no difference between ears (*t*_(26)_ = − 0.8225, *p* = 0.4183). **d** A nonparametric cluster-based permutation analysis indicated an effect of condition for brain power pooled across 0.25–1.95 s of the cue-target interval (*p* = 0.004). This corresponded to a positive cluster in the observed data beginning around 4–6 Hz up to 24–25 Hz. The number of voxels in this cluster is shown as a function of frequency. The extent of the cluster is largest in the alpha and beta bands. Moreover, for both bands, it is located in posterior regions

Next, we tested the hypothesis that cochlear activity is increased during periods of focused auditory compared to visual attention. Descriptively, it appears from the grand average that the amplitude differences (Fig. 2a, b) of the amplitude modulation index (AMI) lie predominantly in the range of low frequencies, corresponding to the frequency range of dominant rhythmic cochlear activity (Fig. 1a, b). Given this overlap, the AMI was pooled across the range of peak frequencies (left ear: 3–10 Hz; right ear: 1–10 Hz) for the cochlear response frequency range of 1000–2000 Hz for the left and right ear, respectively. In the next step, FDR-corrected one-tailed one sample *t* tests against 0 were performed (see Fig. 2c). The result for the left ear revealed that induced cochlear activity (*M* = 1.1002%, *SE* = 0.3047%) was significantly higher for the Attend Auditory condition (*t*_(26)_ = 2.4701, *p* = 0.0122). Similarly, the result for the right ear revealed significantly higher induced cochlear activity (*M* = 1.5343%, *SE* = 0.3047%) for the Attend Auditory condition (*t*_(26)_ = 2.3881, *p* = 0.0122). No interaural differences could be observed (*t*_(26)_ = − 0.8225, *p* = 0.4183). In an analogous manner, we performed FDR-corrected one-tailed one sample *t* tests against 0 for evoked cochlear activity. The results demonstrate that in both ears evoked cochlear activity was not significantly higher for the Attend Auditory condition (left: *t*_(26)_ = − 1.5779, *p* = 0.9367; right: *t*_(26)_ = − 0.6909, *p* = 0.9367). These analyses propose that while induced cochlear activity shows attentional modulations evoked cochlear activity seems not to be modulated by attention.

### Cortical alpha and theta power are related to cochlear changes

In order to assess effects of intermodal attention on brain level, we performed a nonparametric cluster-based permutation analysis on source-projected MEG-power over frequencies of 3–25 Hz (see the “Methods” section). The analysis was pooled across 1.7 s of the cue-target interval. An effect of condition (Attend Auditory > Attend Visual, *p* = 0.004) was observed that corresponded to a positive cluster in the observed data beginning around 4–6 Hz up to 24–25 Hz. As hypothesized, the extent of this cluster is largest in the alpha and beta range and located in posterior—mainly occipital and parietal—brain regions (see Fig. 2d).

We expected inhibited sensory processing of the current task-irrelevant sensory modality—occipital regions for the visual and temporal regions for the auditory modality. According to dominant frameworks [23], this functional inhibition should manifest as increased power in the alpha band. We found increased alpha power for the Attend Auditory condition over occipital regions. However, no increased alpha power for the Attend Visual condition in auditory regions could be found. This absence may be related to a reduced measurement sensitivity due to the significant loss of MEG sensors covering the temporal regions.

**Fig. 3:**
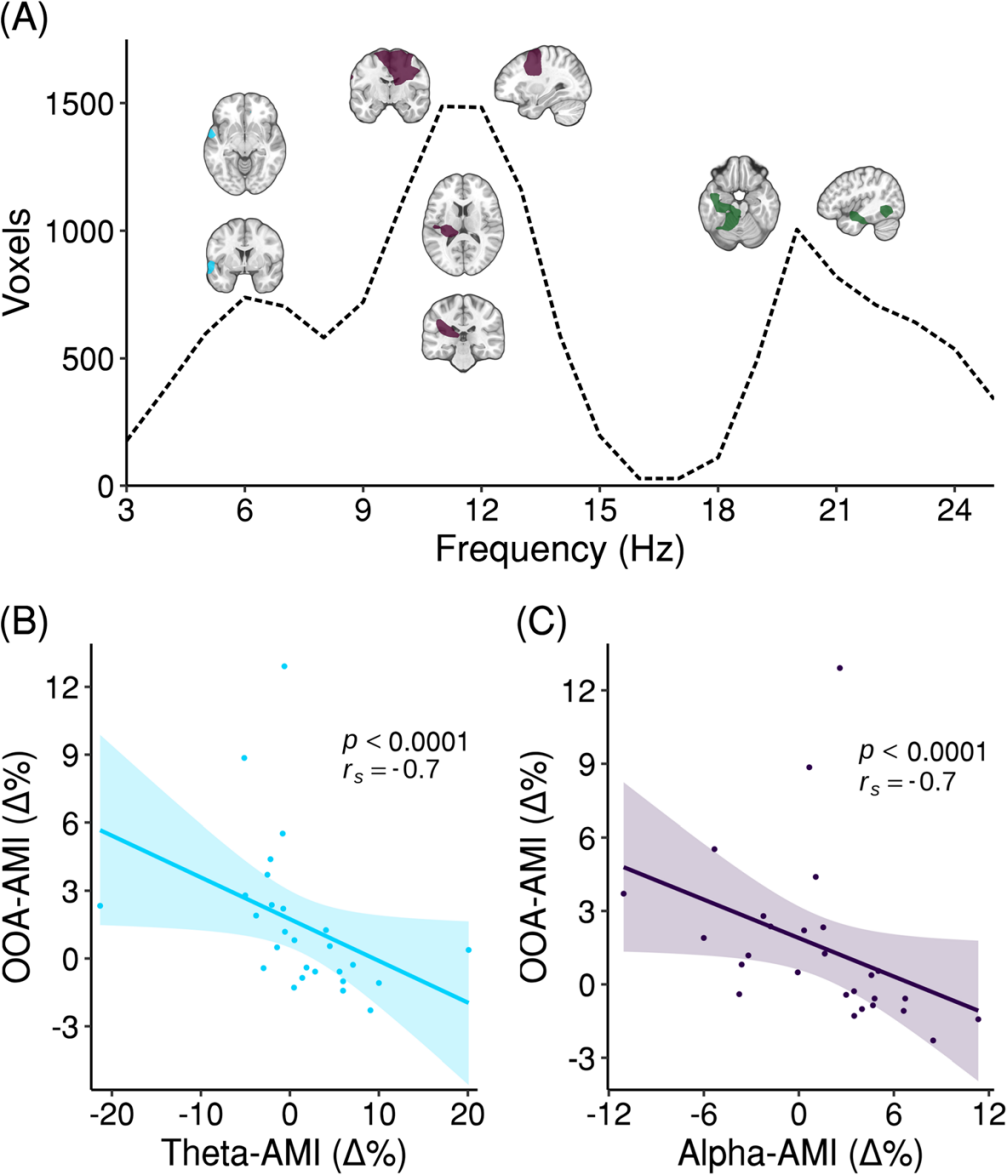
Correlation of cortical neural activity and OOA of the right ear. **a** A nonparametric cluster-based permutation analysis indicated a correlation of brain-AMI and OOA-AMI of the right ear pooled across 0.25–1.95 s of the cue-target interval (*p* = 0.01). This corresponded to a negative cluster in the observed data incorporating the whole frequency range (3–25 Hz) of the analysis. The number of voxels in this cluster is shown as a function of frequency. The extent of the cluster peaks in the alpha, theta, and beta bands. For the peak in the theta band, the cluster is located in the left STG. For the alpha band, it is located in medial portions of left Heschl’s gyrus and right (pre-)motor areas. For the beta band, it is located in left inferior-medial temporal regions. Orthogonal views represent masked *t* values (75% threshold). **b** Correlation of brain-AMI at 6 Hz and OOA-AMI in the most significant voxel from **a**. **c** Correlation of brain-AMI at 11–12 Hz and OOA-AMI in the most significant voxel from **a**. The shaded error bars represent the SEM

In order to assess whether attentional effects found at the cortical level were associated with the previously described cochlear effects, a correlation between the brain-AMI and the induced OOA-AMI of the left (pooled across 3–10 Hz) and right ear (pooled across 1–10 Hz), respectively, was calculated. A nonparametric cluster-based permutation analysis indicated a significant correlation of brain-AMI and OOA-AMI of the right ear (*p* = 0.01) but not the left ear (*p* = 0.62). This corresponded to a negative cluster in the observed data incorporating the whole frequency range (3–25 Hz) of the analysis (see Fig. 3a). The extent of the cluster peaks in the alpha, theta, and beta bands. Dominant locations of the correlation effect are illustrated in Fig. 3a (see Additional file 1: Fig. S1 for an illustration on the brain’s surface). For the theta and alpha frequency range, strong auditory cortical effects are seen in the left STG or medial portions of Heschl’s gyrus, respectively. Interestingly, the effects are strongest contralateral to the OAE probe. However, effects were also observed outside of classical auditory cortical regions, such as in the right (pre-motor) or left inferomedial temporal regions. To illustrate that effects are not driven by outlying participants of relevant effects in the theta and alpha bands, Fig. 3b, c show correlations for voxels with the strongest effects. The negative correlations indicate that lower alpha and theta AMI is accompanied by higher OOA-AMI and vice versa. It is well known that decreasing alpha activity represents a mechanism for a release of inhibition [23, 25]. Thus, the negative correlation suggests that participants exhibiting a stronger release of inhibition (by lower alpha power) in the left auditory brain regions during periods of auditory attention also exhibit elevated OOA-levels (by higher OOA power). This analysis illustrates that attentional modulations of rhythmic activity at the “lowest” (i.e., cochlear) level of the corticofugal system go along with modulations of oscillatory brain activity at the “highest” level. The absence of a significant effect for the correlation with the OOA-AMI of the left ear could be explained by the high amount of saturated sensors in (contralateral) temporal regions, which is caused by magnetic artifacts of the microphone probes (see the “Methods” section). Depending on the number of bad sensors on each side measurement sensitivity can be severely reduced in respective temporal regions.

### OOA is not sensitive to within-subject performance variability

Finally, we investigated if the OOA- and cortical effects were sensitive to within-subject performance variability as these kinds of analyses provide more insight into how attention modulates both cortical and cochlear activity. Since accuracies show a ceiling effect, analyses are exclusively run for reaction times. So, for each subject and condition, trials were individually split into slow and fast trials by median splits. In performing the median split for each subject and condition individually, we avoid confounding effects of between-subject and intermodal performance variability.

Initially, analyses for the induced OOA were calculated. A three-factorial ANOVA (2 × 2 × 2) with the repeated measures factors ear (left and right), reaction time (slow and fast), and condition (auditory and visual) was calculated for peak frequencies. The results revealed no significant main effects (ear: *F*_(1, 26)_ = 0.8273, *p* = 0.3714; reaction time: *F*_(1, 26)_ = 0.3177, *p* = 0.5778; condition: *F*_(1, 26)_ = 1.4210, *p* = 0.2440). Next, the same ANOVA was calculated for slopes. Again, its results revealed no significant main effects (ear: *F*_(1, 26)_ = 0.3452, *p* = 0.5619; reaction time: *F*_(1, 26)_ = 2.7340, *p* = 0.1103; condition: *F*_(1, 26)_ = 0.2272, *p* = 0.6376).

Subsequently, a two-factorial ANOVA (2 × 2) with the repeated measures factors ear (left and right) and reaction time (slow and fast) was calculated for induced OOA-AMIs. The results showed no significant main effects (ear: *F*_(1, 26)_ = 1.0540, *p* = 0.3141; reaction time: *F*_(1, 26)_ = 1.6380, *p* = 0.2119). As there is no “one-sample ANOVA,” we additionally performed FDR-corrected one-tailed one sample *t* tests against 0 to test if the OOA-AMI in slow and fast trials is increased during periods of focused auditory attention. The results revealed that the OOA-AMI in slow trials of the left ear (*M* = 0.0631%, *SD* = 4.4203%) was not significantly increased in the auditory condition (*t*_(26)_ = 0.0741, *p* = 0.4828). The OOA-AMI in slow trials of the right ear (*M* = 0.0399%, *SD* = 4.7708%) failed to be significantly increased in the auditory condition (*t*_(26)_ = 0.0435, *p* = 0.4828). The same pattern was found for fast trials in both ears. The OOA-AMI in fast trials of the left ear (*M* = 1.8846%, *SD* = 4.6601%) was not significantly increased in the auditory condition (*t*_(26)_ = 2.1010, *p* = 0.0661). The OOA-AMI in fast trials of the right ear (*M* = 2.8994%, *SD* = 7.8528%) was not significantly increased in the auditory condition (*t*_(26)_ = 1.9190, *p* = 0.0661).

To assess effects of reaction times (slow vs. fast trials) on brain level, we performed a nonparametric cluster-based permutation analysis on source-projected MEG-power over frequencies of 3–25 Hz (see the “Methods” section). The analysis revealed no effect of pretarget MEG-power on reaction times.

Overall, the reported result for cortical activity does not indicate a sensitivity to reaction times. The same holds true for cochlear activity. However, the OOA-AMI in fast trials just fails to be significantly higher for auditory attention compared to visual attention.

## Discussion

To what extent cochlear activity is sensitive to selective attention and how these changes are linked to cortical dynamics is a matter of ongoing debate. Given the uniqueness of the auditory system in having cortical descending projections from primary auditory cortex (via IC and SOC) to the cochlea, it is conceivable that a putative mechanism of alternating attentional states directly affecting cochlear processes could exist. To pursue our aims, we adapted a previously introduced approach for investigating cochlear otoacoustic activity [20] that allows us to draw first conclusions on how cortical attention processes are linked to cochlear otoacoustic activity. We demonstrate the presence of a theta-rhythmic pattern of otoacoustic activity during silent periods when attention was focused on either upcoming auditory or visual targets. Furthermore, we established a relationship between cochlear theta and cortical alpha modulations during the cue-target intervals. Despite several open issues remaining, this study creates a connection between cochlear and cortical attentional modulations and helps close the gap between the remarkably segregated auditory attention research lines.

Our analysis of the OOA during the cue-target interval indicated a genuine rhythmic modulation in the theta frequency range (~ 6 Hz on average) that was not explicable by aperiodic (“1/f”) contributions to the spectrum. Furthermore, this theta rhythm was not phase-aligned across trials. The peak frequency of the found rhythmic OOA pattern does not differ between visual and auditory attention nor slow and fast trials, indicating the existence of a general endogenous cochlear rhythm at ~ 6 Hz. This finding is in line with Ho et al. [26] that applied signal detection theory to test for oscillations of behavioral performance in a bilateral pitch-identification task. They reported for both sensitivity and criterion strong oscillations over time where sensitivity oscillated at ~ 6 Hz and criterion at ~ 8 Hz. Thus, it is conceivable that sensory input from both ears is rhythmically sampled in the theta band (3–8 Hz). Interestingly, we also found a slower evoked oscillation at ~ 4 Hz. However, the peak frequency and phase of this component did not differ between visual and auditory attention. Moreover, in contrast to Ho et al. [26], the phase of the evoked ~ 4 Hz oscillation was not different between ears. Depending on the generating mechanisms of the theta rhythmic cochlear activity, perceptual or attentional rhythmicities could either be genuine cortically driven effects (with cochlear effects being epiphenomenal) or they (and by extension cortical effects) could be an adaptation to cochlear physiological processes. However, the interindividual difference in peak frequencies was rather high and primarily encompasses the theta and alpha bands. The involvement of both the theta and alpha bands hints at different mechanisms (e.g., theta as a sampling mechanism and alpha as an inhibition mechanism) that putatively contribute to attention processes on the cochlea. This assumption is backed by the active sampling [27] literature, which points to the ubiquitousness of theta-like rhythms in various cognitive domains ranging from perception to action [28–31]. Extending such views, a recent “rhythmic theory of attention” framework states that attention is theta-rhythmically discontinuous over time [32–35]⁠. While the latter framework has been developed mainly to better understand visuospatial attention, similar processes may also be relevant in the auditory system. For example (not in the focus of the current study), it is conceivable that interaural attention modulates the phase of the theta rhythm in both ears, facilitating signal transduction in the to-be-attended ear.

Beyond the illustration of a slow (theta) rhythmic modulation of OOA during silent cue-target intervals independent of the attention focus, we show that the magnitude of this process is clearly attentionally modulated for induced but not evoked power. We found an enhancement during auditory selective attention, which was not sensitive to reaction times and might reflect an enhancement of cochlear sound amplification. In line with previous studies that found reduced levels of OAEs in subjects attending to a visual task, our results resemble an elevation of the to-be-attended acoustic stimulus during acoustic selective attention ([13, 16, 35, 36]; see [11, 37]⁠ for an exception). Particularly, one study consistently reported similar amplitude modulations at low frequencies (< 7 Hz [20];). Yet, thus far, all studies on humans that have investigated effects of attention on the cochlea in cue-target intervals utilized different types of evoked OAEs (EOAE) and distortion product OAEs (DPOAE)⁠. The measurement of EOAEs and DPOAEs relies on acoustic elicitor and probe stimuli, which are able to alter cochlear properties by themselves, making them rather unfavorable for assessing pure efferent effects [19]. It has to be noted that there are two studies that also investigated effects of attention (auditory and visual) and inattention on the cochlea by measuring physiological noise in a silent period subsequently of evoking nonlinear stimulus-frequency OAEs [38, 39]. However, both studies differ from the current one as they analyzed cochlear activity after stimulation and did not compare auditory and visual attention effects. In our study, we utilized OOA that is measured in silent cue-target intervals and therefore avoids any confounding efferent activity. Moreover, our approach allows us to stay as close as possible to previous literature in the cortical attention domain. In the current study we show power modulations of OOA in frequencies that in the cortical literature have been repeatedly reported to be related to various attentional task demands [23, 33, 35, 40]. Electrical stimulation of the auditory cortex in bats and chinchillas shows that cochlear responses can be modulated in a frequency specific manner [41–43]. The current results imply that the modulation of cochlear low-frequency oscillatory power putatively is driven by top-down attentional processes (note that the frequency is unchanged). Given the well-established neuroanatomy of the auditory efferent system, corticofugal projections from the auditory cortex to the cochlear receptor, which are mediated by the IC and SOC, are the most probable neural substrates of this effect. The correlation effects of the present study are compatible with this interpretation.

The current results of induced oscillatory activity in the MEG are in accordance with previous results and give an insight into the attentional demands of the task. Despite the unfavorable measurement conditions, we found elevated alpha and beta band activity in the pretarget period of Attend Auditory compared to Attend Visual trials in posterior regions but no modulations over auditory regions. Various studies on intermodal selective attention have postulated an active role of cortical alpha oscillations in modulating primary sensory areas [13, 17, 18, 22, 44]. In this context, alpha band activity is proposed to reflect a suppression mechanism and especially seems to be relevant if distracting input has to be actively blocked. Two studies employing an audiovisual task have reported alpha power increases in posterior sensors when attention was directed to the auditory modality, power decreases when attention was directed to the visual modality, and no alpha band modulations over auditory cortices [17, 22]. In line with these findings, Wittekindt et al. [13]⁠ observed a relative posterior alpha power increase when attention was focused on the upcoming auditory compared with the visual target. Our findings showing increased alpha power in the primary visual cortex during auditory selective attention are in accordance with this view. In this way, alpha oscillations act to reduce processing of distracting input for the task-irrelevant visual modality.

Three previous studies have simultaneously recorded DPOAEs and EEG and were therefore able to investigate the relationship between cochlear and brain activity. Wittekindt et al. [13] failed to show any correlations between those two. The authors explain this by the fact that their found effects depict different mechanisms of selective attention and thus do not depend on each other directly. In contrast, Dragicevic et al. [20]⁠ reported significant correlations between the oscillatory DPOAE signal and cortical oscillations at low frequencies (< 10 Hz) mainly when attention was switched from the visual to the auditory modality. Finally, studying predictive processing using an intermodal predictability paradigm Riecke et al. [45] found a relationship between DPOAE and brain effects. However, this relationship is limited to participants that benefited from predictions. Overall, as mentioned above, the elicitor stimuli which are required to evoke DPOAEs are prone to elicit MOC efferent activity that causes intrinsic cochlear changes by themselves. Hence, any inferences from correlations between oscillatory activity of the cochlea and the brain have to be treated with caution. The current study avoids these pitfalls by utilizing OOA in silent periods.

We found evidence for a putative relationship, namely, a negative correlation of induced cochlear low-frequency (1–10 Hz) power of the right ear and brain power, during periods of selective attention. This correlation was especially pronounced in the alpha, theta, and beta bands and was located in left auditory processing regions. It appears that subjects that exhibit a stronger cortical release of inhibition of auditory input (by reduced alpha power) at the same time show stronger enhancement of the auditory target in the auditory periphery (by enhanced low-frequency OAA-power) and vice versa. Furthermore, the correlation in the theta band is strongest at ~ 6 Hz, the same frequency as the extracted periodic component of the induced OOA. Taking the relationships in the alpha and theta bands together, they could point to a mechanism for a release of inhibition. Considering the architecture of the auditory efferent system, it is likely that the outlined auditory cortical regions are a departure point for top-down modulations of cochlear activity in the current experiment. The observed cortico-cochlear correlations are compatible with the notion that these top-down modulations propagate through the efferent auditory pathway via crossed MOC fibers [8]. Interindividual variability appears to exist to the extent that this top-down modulation is deployed next to the predominant inhibition of visual processing regions. In accordance with our findings (see also [17]), we suggest that top-down control of cochlear processing by cortical regions is mediated by slow oscillatory brain activity.

The current study did not utilize threshold-level stimuli, which resulted in a ceiling effect for accuracy. In turn, it cannot be ruled out that subjects potentially were able to attend to both modalities. Yet, such a behavior would not yield any behavioral advantages in the current task. Nevertheless, we cannot exclude that subjects were able to attend to both modalities and, in turn, that results possibly could be biased by divided attention processes. Future studies should parametrically vary task difficulty to investigate this issue.

## Conclusion

The present study implies the existence of an putatively endogenous cochlear rhythm in the theta band—a rhythm suggested to be linked to active sampling of the environment in different modalities [27, 32, 34]. An outstanding question for future research is to understand the mechanistic relationship between cochlear theta rhythms and—especially auditory—cortical rhythms. Our results show that cochlear activity is modulated by intermodal top-down attention. In this regard, it provides evidence for the ongoing debate, whether the human auditory periphery is sensitive to top-down modulations [6, 8]. Future studies should investigate if rhythmic auditory processing is present even in the absence of stimulus input or predictable targets (e.g., at “resting state”) and how these processes are manifested in individuals with reported hearing problems with or without audiometric deficits.

## Methods

### Participants

Thirty-four healthy volunteers (23 females, age range 18–35 years) participated in this study. One participant was excluded from analyses because his right ear was occluded by cerumen. As recording otoacoustic activity inside an MEG system is challenging, further participants were also excluded from the final analysis (see in the “Results” section for details). One participant was excluded because the left acoustic meatus was too small to fit the foam ear tip without causing pain. One participant was excluded because the recordings from the left ear showed excessive periods of saturation. Another four participants were excluded because the number of artifact contaminated MEG trials exceeded two standard deviations of the mean. The remaining 27 volunteers (18 female, mean age 22.96 years, age range 18–35 years) were used for analyses. Four participants were left handed. None of the participants reported any known hearing deficit and any visual impairment was corrected to normal with MEG-compatible glasses. All subjects were informed about the experimental procedure and the purpose of the study and gave written informed consent. As compensation, subjects received either €10 per hour or credit for their psychology studies. This study was approved by the Ethics Committee of the University of Salzburg.

### Stimuli and procedure

Our focus in this study was to investigate intermodal selective attention by simultaneously measuring cochlear (OOA) and neuronal processes (MEG). Studies investigating attentional modulations of OAEs in the past often used a block design [11, 36, 46]. As this procedure is criticized for not achieving highly controlled attentional conditions [13, 47, 48], we decided to use an adapted version of the trial-wise cueing paradigm introduced by Wittekindt et al. [13].

**Fig. 4:**
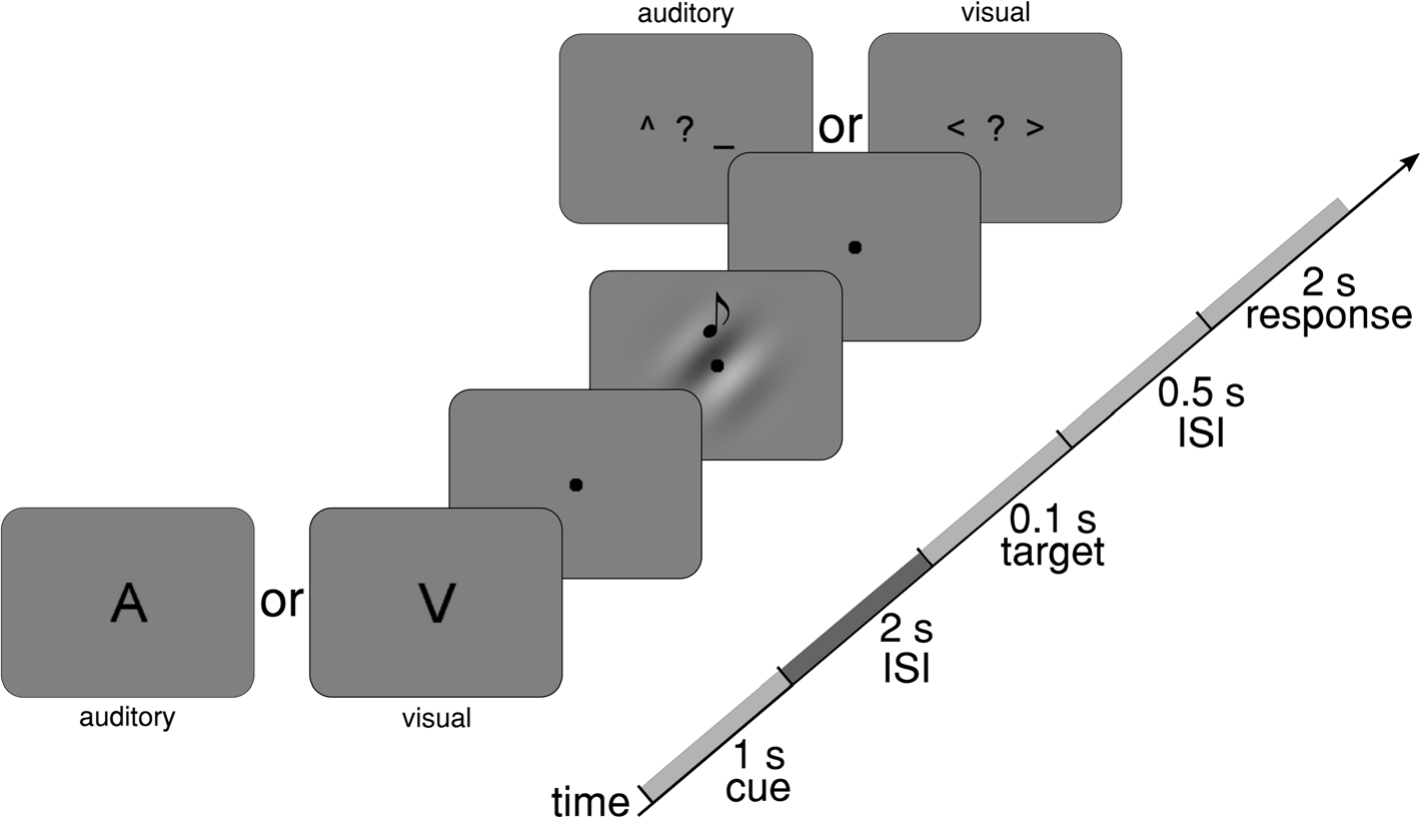
Schematic illustration of the task. Each trial started with a 100% informative visual cue telling the subject to either attend the auditory (“A”) or the visual modality (“V”). After an ISI of 2 s, a left- or right-oriented Gabor patch and a low-frequency (1131 Hz) or high-frequency (1987 Hz) pure tone were simultaneously presented. After another ISI of 0.5 s, a response screen depending on the cued modality appeared for 2 s. The intertrial interval was uniformly jittered between 1 and 2 s

Measurements took place in a magnetically shielded room (AK3B, Vacuumschmelze, Hanau, Germany), in which subjects sat quietly inside the MEG system (TRIUX, MEGIN-Elekta Oy, Helsinki, Finland). Participants performed five blocks consisting of 80 trials (40 Attend Auditory and 40 Attend Visual) in a pseudo-randomized order. Figure 4 schematically illustrates the course of a trial. Each trial started with a visually presented cue (1 s duration) instructing the subject to either attend the auditory or the visual modality. The letter “A” indicated the Attend Auditory condition and the letter “V” the Attend Visual condition. During the following silent cue-target interval (2 s duration), a fixation dot was presented and the participants had to shift their attention selectively to the indicated modality. To reduce effects of divided attention and to reach maximum focus on the cued modality, the cue was 100% informative [13]. The target stimulus in the visual modality was a low-contrast Gabor patch (diameter: ca. 2° of visual angle) that was displayed in the center of a rear projection screen placed inside the shielded room (distance to the subject 1.1 m) and oriented 45° to the right or left. The target stimulus in the auditory modality was a pure tone of either 1131 Hz or 1987 Hz, which was presented via ear inserts. The sound volume was individually adjusted to be at a comfortable level. Visual and auditory stimuli were simultaneously presented for 100 ms. For the auditory stimuli, we employed two 5 ms linear fade in/out windows. Depending on the preceding cue, the task was to detect the orientation of the Gabor patch (Attend Visual, left or right 45° tilt) or the pitch level of the tone (Attend Auditory, high pitch (1987 Hz) or low pitch (1131 Hz)). Afterwards, a response screen showed indicators for choosing either the pitch level of the tone or the orientation of the Gabor patch. Participants were instructed to wait until the response screen was presented (0.5 s post-target), and then reply as soon as they were ready by pressing the corresponding button with their left or right thumb, within 2 s after the appearance of the response screen. The inter-trial intervals were jittered uniformly between 1 and 2 s. Acoustic and visual stimuli were generated by the Psychophysics Toolbox Version 3 [49, 50] using custom-written MATLAB scripts (Version 9.1; The MathWorks).

### Recording of cochlear and cortical activity

In order to measure otoacoustic activity, a probe consisting of a sensitive microphone and two loudspeakers (ER-10C microphone/preamplifier system, Etymotic Research, Elk Grove Village, USA) was fitted into the subject’s right and left ear canal with a foam ear tip. Otoacoustic activity was recorded from both ears concurrently. The microphone signal was fed into the EEG amplifier of the MEG system, with an amplitude gain of + 55 dB (600x). The sampling rate of the entire MEG and EEG system was set to 10 kHz. The ER-10C received its input via two BNC cables coming from a sound preamplifier (SOUNDPixx, VPixx Technologies, Saint-Bruno, Canada). The SPL for the loudspeakers was balanced to the left and right side by subjective feedback for each participant.

Neuromagnetic brain activity was recorded with 306 channels (TRIUX MEG, see above). Two bipolar electrodes were mounted above and below the left eye: one was mounted on the left side of the left eye and another on the right side of the right eye to monitor eye blinks and eye movements (H/VEOG). Further, two electrodes were mounted on the bottom left rib and the right collarbone to record electrocardiography (ECG). A reference electrode was placed on the left trapezius muscle and the ground electrode on the right supinator. Prior to the experiment, individual head shapes were acquired for each participant including relevant anatomical landmarks (nasion and preauricular points) and about 300 digitized points on the scalp with a 3D digitizer (Polhemus FASTRAK, Colchester, US). Head positions of the subjects in the helmet were estimated at the beginning of each block injecting a small current into five (HPI, head position indicator) coils. Again, the overall (MEG + EEG) sampling rate was set to 10 kHz, with a hardware high-pass filter of 0.1 Hz, and an anti-alias low-pass filter with the cutoff frequency set to 3330 Hz.

### Signal processing

OOA was preprocessed by high-pass filtering at 500 Hz (6th order Butterworth IIR), extracting epochs of 3 s duration after cue presentation and manually rejecting trials containing periods of signal saturation or atypical high background noise, for example, caused by moving, swallowing, or coughing (average number of rejected trials per participant: 87.15; range across participants 1–185). As the frequencies of the acoustic targets were between 1131 Hz and 1987 Hz and otoacoustic activity is strongest in the range from 1000 to 2000 Hz [51], we expected amplitude modulations of the OOA in this range. The cue-target interval was defined as the period in which intermodal attention processes occur [13]. In the next step, trials were split into two conditions (Attend Auditory and Attend Visual), averaged over 1.7 s of the cue-target interval, and bandpass filtered in 10 Hz steps from 1000 to 2000 Hz (bandpass window ± 30 Hz). This resulted in 201 bandpass windows for each participant, which represent the binned cochlear frequency response between 1000 and 2000 Hz. To be able to further study any relationship between cochlear activity and brain oscillations (see the “Results” section), we extracted the envelope of the cochlear signal for each of the previous bandpass windows via a Hilbert transform, thus obtaining a signal with a frequency range that is routinely used in electrophysiological evaluations of cognitive tasks. Next, induced PSD from 1 to 30 Hz was calculated for each condition and each Hilbert transformed bandpass window (“mtmfft” fieldtrip implementation with a Hann window). Finally, the bandpass windows were concatenated for each condition resulting in a representation of the amplitude modulation from 1 to 30 Hz at cochlear response frequencies from 1000 to 2000 Hz.

The MEG signal was first preprocessed by manually rejecting all bad sensors (average number of rejected sensors per participant 38.89; range across participants 13–73), high-pass filtering at 1 Hz (6th order Butterworth IIR), extracting epochs of 3 s duration after cue presentation, and down-sampling to 1 kHz. The excessive amount of rejected sensors is caused by magnetic artifacts of the microphone probes, which leads to a saturation of several mostly temporal sensors. The detected bad trials in the OOA data were used to reject the same trials in the MEG data. In the next step, trials were again split into two conditions (Attend Auditory and Attend Visual). For source level analysis, a standard anatomical magnetic resonance imaging (MRI) template provided by the Statistical Parametric Mapping toolbox (Version 12; [52]) was morphed to the individual head shape of each participant using non-linear-transformation. Sensor space trials were projected into source space using linearly constrained minimum variance (LCMV) beamformer filters [53]. The aligned brain volumes were also used to create single-shell head models and compute the leadfield matrices [54]. For the template grid, we chose a resolution of 1 cm in MNI space. Induced PSD in 1 Hz steps in a frequency range of 1–30 Hz averaged over 1.7 s of the cue-target interval was calculated for each condition by a FFT (Hann window). The preprocessing of the OOA and MEG data was conducted using the open-source FieldTrip toolbox for EEG/MEG data [55] and custom-written MATLAB scripts (Version 9.1; The MathWorks).

### Statistical analysis

As a first analysis step, we investigated if rhythmic modulations of cochlear activity are present. The python (Version 3.7.1) toolbox FOOOF [56]⁠ was used to parameterize the induced and evoked power spectra of the OOA envelope of each subject and condition. FOOOF allows for the examination of putative oscillations (peaks) in the frequency domain and characterizes these on their specific center frequencies, amplitude, and bandwidth by separating the periodic and aperiodic components of neural power spectra [56]⁠. The separation of periodic and aperiodic components is the key feature of the FOOOF-toolbox and allows the characterization of putative oscillations without aperiodic contributions. In order to evaluate if the identified peaks are significant components of each PSD, we tested if the power at each peak frequency is a significant outlier of the power distribution at all frequencies that are not identified as a peak by using Dixon’s *Q* tests. Next, we tested if the proportion of significant results of Dixon’s *Q* tests was significantly different from chance by using an exact binomial test. Subsequently, we performed Kolmogorov-Smirnov tests to test for uniformity on the peak frequencies for every ear and condition. A significant deviation from uniformity suggests that the peaks were not identified by chance. Finally, we calculated circular common median tests to investigate if the phase of the evoked oscillation differs between ears and modalities.

For statistical analyses of the periodic components of the induced OOA, the attention modulation index (AMI) of both conditions was calculated using the following formula: (Attend Auditory – Attend Visual)/(Attend Auditory + Attend Visual) × 100. A one-tailed one sample *t* test against 0 for each ear was calculated for the induced and evoked AMI pooled across the full range of the cochlear frequency response (1000–2000 Hz) and the range of extracted peaks from the left and right ear. A nonparametric cluster-based permutation analysis over the whole brain was conducted to assess MEG-power effects in the cue-target interval. The analysis was pooled across 1.7 s of the cue-target interval and limited to a frequency range of 3–25 Hz. In the next step, the AMI of the MEG-data was calculated and correlated with the induced OOA-AMI of the left and right ear. In order to assess statistical significance of the correlation, a nonparametric cluster-based permutation analysis over the whole brain was conducted. As for the assessment of MEG-power effects, this analysis was pooled across 1.7 s of the cue-target interval and limited to a frequency range of 3–25 Hz.

Finally, statistical analyses to assess effects of within-subject performance variability were conducted. So, for each subject and condition, trials were split into slow and fast trials by median-splits. Three-factorial ANOVAs (2 × 2 × 2) with the repeated measures factors ear (left and right), reaction time (slow and fast), and condition (Auditory and Visual) were calculated for induced peak frequencies and slopes. Subsequently, a two-factorial ANOVA (2 × 2) with the repeated measures factors ear (left and right) and reaction time (slow and fast) was calculated for induced OOA-AMIs. We calculated one-tailed one sample *t* tests against 0 for each ear to test if the OOA-AMI in slow and fast trials is increased during periods of focused auditory attention. To assess effects of reaction times (slow vs. fast trials) on brain level, we performed nonparametric cluster-based permutation analyses on source-projected MEG-power over frequencies of 3–25 Hz and pooled across 1.7 s of the cue-target interval.

For all statistical analyses that required a correction for multiple comparisons, we corrected the *p* values using the FDR method set at 0.05. The statistical analyses of the OOA and MEG data were conducted using the open-source FieldTrip toolbox for EEG/MEG data [55], the “CircStat for Matlab”-toolbox [57], custom written MATLAB scripts (Version 9.1; The MathWorks), the R packages “uniftest: Tests for Uniformity” [58], “outliers” [59], and custom written R scripts (Version 4.0.0; R Core Team).

## Supplementary Information

**Additional file 1: Figure S1.** The same data as in Fig. 3A is illustrated but on the brain’s surface instead of orthographic slices.

## Data Availability

The datasets analyzed during the current study are deposited under https://zenodo.org/record/4389203 [60].
