# Supplementary figures and images for: Cochlear activity in silent cue-target intervals shows a theta-rhythmic pattern and is correlated to attentional alpha and theta modulations
Source: BMC Biol. 2021 Mar 16;19:48. doi: 10.1186/s12915-021-00992-8 (PMC7968255; doi:10.1186/s12915-021-00992-8)

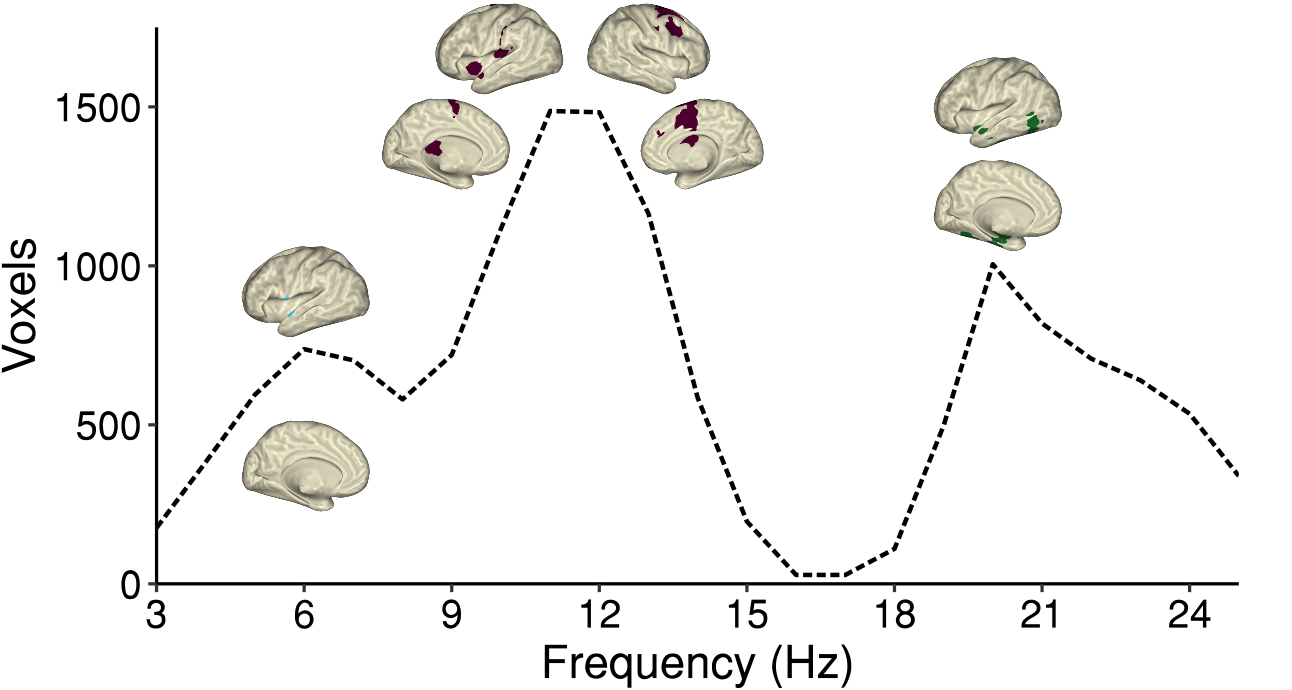

Supplement: Supplementary file 1 — Additional file 1: Figure S1. The same data as in Fig. 3A is illustrated but on the brain’s surface instead of orthographic slices. [file 12915_2021_992_MOESM1_ESM.png]
